# Reduced SERCA activity underlies dysregulation of Ca^2+^ homeostasis under atmospheric O_2_ levels

**DOI:** 10.1096/fj.201700685RRR

**Published:** 2018-01-08

**Authors:** Thomas P. Keeley, Richard C. M. Siow, Ron Jacob, Giovanni E. Mann

**Affiliations:** King’s British Heart Foundation Centre for Research Excellence, School of Cardiovascular Medicine and Sciences, Faculty of Life Sciences and Medicine, King’s College London, London, United Kingdom

**Keywords:** Ca^2+^ signaling, physiological normoxia, oxygen, mitochondria, Ca^2+^ overload

## Abstract

Unregulated increases in cellular Ca^2+^ homeostasis are a hallmark of pathophysiological conditions and a key trigger of cell death. Endothelial cells cultured under physiologic O_2_ conditions (5% O_2_) exhibit a reduced cytosolic Ca^2+^ response to stimulation. The mechanism for reduced plateau [Ca^2+^]_i_ upon stimulation was due to increased sarco/endoplasmic reticulum Ca^2+^ ATPase (SERCA)-mediated reuptake rather than changes in Ca^2+^ influx capacity. Agonist-stimulated phosphorylation of the SERCA regulatory protein phospholamban was increased in cells cultured under 5% O_2_. Elevation of cytosolic and mitochondrial [Ca^2+^] and cell death after prolonged ionomycin treatment, as a model of Ca^2+^ overload, were lower when cells were cultured long-term under 5% compared with 18% O_2_. This protection was abolished by cotreatment with the SERCA inhibitor cyclopiazonic acid. Taken together, these results demonstrate that culturing cells under hyperoxic conditions reduces their ability to efficiently regulate [Ca^2+^]_i_, resulting in greater sensitivity to cytotoxic stimuli.—Keeley, T. P., Siow, R. C. M., Jacob, R., Mann, G. E. Reduced SERCA activity underlies dysregulation of Ca^2+^ homeostasis under atmospheric O_2_ levels.

It is well established that routine cell culture under standard conditions (18% O_2_ at sea level) is far removed from the physiologic milieu with respect to O_2_ (1, 2). In comparison with the culture of cells under physiologic (3–5%) O_2_, a significant impact of hyperoxic culture has been demonstrated in macrophages (3), endothelial cells (ECs) (4–6), stem cells (7), and leukocytes (8). We have recently described the consequences of long-term culture of ECs under physiologic (5%) O_2_ levels on nuclear factor E2–related factor 2 (4) and NO signaling (6).

The influence of O_2_ on Ca^2+^ signaling has been widely studied in specialized O_2_-sensing cells such as those in the carotid body and pulmonary arteries (2). These cells possess O_2_-sensitive channels (Kv1.2/5) through which a rapid and sustained inward current is detectable upon reduction in cytosolic O_2_ (9). In the absence of Kv1.2/5 channel expression, the influence of O_2_ on Ca^2+^ signaling in nonexcitable cells is more complex. During acute hypoxia (1–3% O_2_), increased influx through the Na^+^:Ca^2+^ exchanger (NCX) has been demonstrated in HUVECs (10) and chondrocytes (11), whereas a verapamil-sensitive influx has been observed in mesangial cells (12). Reactive oxygen species–mediated alterations in the sensitivity of ryanodine receptors also contribute to changes in Ca^2+^ homeostasis in saphenous vein ECs (13) and C2C12 skeletal muscle microtubules (14) under hypoxic conditions. We have previously reported reduced plateau [Ca^2+^]_i_ in response to agonist stimulation in HUVECs cultured at 5% O_2_, conditions under which no change in basal redox phenotype is detectable (4). In this study, we characterize the mechanisms underlying this reduced plateau [Ca^2+^]_i_ and provide evidence that physiologic normoxia enhances cytoplasmic Ca^2+^ clearance *via* the sarco/endoplasmic reticulum Ca^2+^ ATPase (SERCA), protecting ECs against Ca^2+^-induced cell death.

## MATERIALS AND METHODS

### Culture of human primary ECs

Umbilical cords were obtained from St. Thomas’ Hospital, and venous ECs were isolated using a collagenase solution. HUVECs were cultured in M199 + 20% fetal calf serum and used at passage 3. Culture under physiologic (5%) O_2_ levels was achieved by transferring cells from a standard CO_2_ incubator (∼18% O_2_) to an atmosphere-regulated workstation set to 5% O_2_ and 5% CO_2_ (Sci-tive; Baker-Ruskinn, Sanford, ME, USA). Cells were adapted to 5% O_2_ from passage 2 for a minimum of 5 d, and subculture was performed at 5% O_2_ with all media and buffers pre-equilibrated to this O_2_ level.

### Intracellular Ca^2+^ measurements

Measurement of intracellular Ca^2+^ was performed as described previously (6). Briefly, HUVECs cultured in 96-well plates were loaded with 2 µM/L fura-2 AM or Fura-PE3 AM for 30 min at 37°C. Fluorescence was measured using a plate reader (CLARIOstar; BMG Labtech, Aylesbury, United Kingdom) at 340 and 380 nm excitation and 510 ± 10 nm emission, and the ratio of 340:380 used to calculate [Ca^2+^]_i_. Where appropriate, the plate reader atmosphere was maintained at 5% O_2_ using an atmosphere control unit. To determine Ca^2+^ influx, add-back and Mn^2+^ quench assays were performed as previously described in detail (15), but adapted to plate reader format.

### Immunoblotting

Immunoblot analysis of protein expression was carried out as described previously (6) with minor modifications. Cell protein lysates were obtained in SDS lysis buffer, and equal amounts of protein [10 µg for SERCA, 50 µg for phospholamban (PLB)] were resolved by electrophoresis (150 V for 2 h on 8–15% SDS gels). Samples for PLB measurements were not boiled to better preserve the pentameric structure (detected at ∼25 kDa). Once resolved, proteins were transferred onto PDVF membranes by semidry transfer at 20 V for 2 h, and protein expression was determined using primary and secondary antibodies as previously described. SERCA2 specific antibodies were kindly provided by Kalwant Authi (King’s College London). Phospho (S16+T17)-PLB antibody was from Abcam (Cambridge, United Kingdom), total PLB antibodies were from Badrilla (Leeds, United Kingdom), and β-actin antibody was from Sigma-Aldrich (Irvine, United Kingdom).

### Measurements of cell viability

Cell viability was assessed using either the 3-(4,5-dimethylthiazol-2-yl)-2,5-diphenyltetrazolium bromide (MTT) assay or annexin V fluorescence. Briefly, cells were incubated with 0.5 mg/ml MTT in M199 for 4 h after treatment. The resulting formazan crystals were dissolved in DMSO, and absorption was measured at 562 nm using a plate reader (Clariostar; BMG Labtech). Results are expressed relative to vehicle (DMSO, 0.01%) treatment. To assess annexin V staining, HUVECs were stained using a CFM640-conjugated annexin V label (Biotium, Cambridge, United Kingdom) with Hoechst 33342 (Sigma-Aldrich) used to counterstain nuclei. Images were obtained using an Orca-03G camera (Hamamatsu Photonics, Hamamatsu City, Japan) and quantified using ImageJ software (National Institutes of Health, Bethesda, MD, USA).

### Data handling and statistical analysis

The raw traces of [Ca^2+^]_i_ are representative of results obtained from at least three different donors. In the figures, data in bar graphs report the means ± sem responses from at least three different donors, where the mean from three to four replicate wells represents a single value for each donor (the quoted n is the number of donors). Significance was assessed using a paired Student’s *t* test or ANOVA where appropriate. A value of *P* < 0.05 was considered significant.

## RESULTS

### Ca^2+^ mobilization in ECs adapted to physiologic O_2_

**Figure 1.**
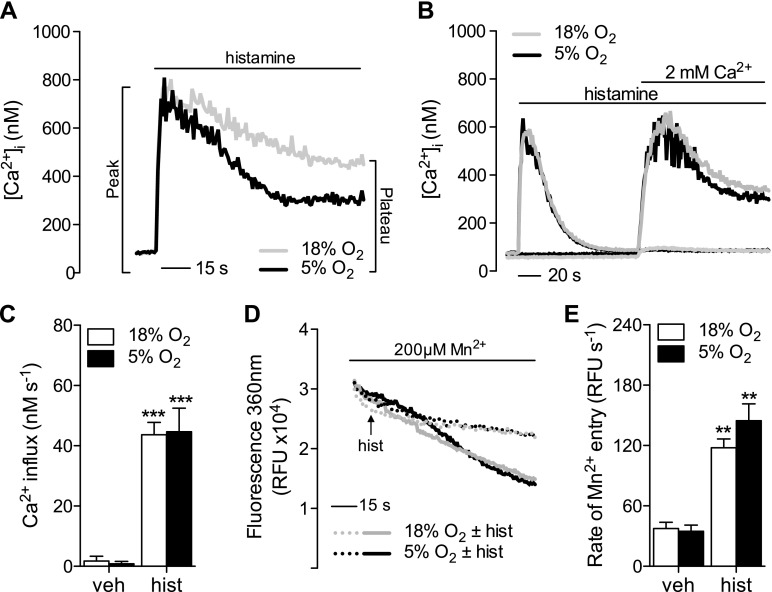
Altered plateau [Ca^2+^] is not due to reduced Ca^2+^ influx. HUVECs were cultured under standard (18%) or physiological (5%) O_2_ levels, and changes in [Ca^2+^]_i_ were determined using fura-2. *A*) Representative response to 10 µM histamine (hist) in the presence of 2 mM external Ca^2+^. *B*) Ca^2+^ influx in response to histamine as determined by the Ca^2+^ add-back assay. Cells were stimulated with histamine or H_2_O vehicle (veh) in nominally Ca^2+^-free buffer, followed by readdition of 2 mM Ca^2+^. A representative trace is shown. *C*) Mean ± sem (*n* = 5–8) rate of Ca^2+^ influx determined by linear regression analysis of the initial rise in [Ca^2+^]_i_ upon readdition of external Ca^2+^. Mn^2+^ (200 µM) influx is shown as a proxy for Ca^2+^, measured as the rate of decrease in emitted fura-2 fluorescence when excited at 360 nm in the presence of 2 mM external Ca^2+^. *D*) Representative trace. The arrow denotes addition of histamine (10 µM). *E*) Summary of the means ± sem from 3 different donors. RFU, relative fluorescence units. **P < 0.01, ***P < 0.001 *vs.* control.

We have previously shown that culture at 5% O_2_ significantly lowers plateau [Ca^2+^]_i_ in human ECs (*i.e.*, HUVECs and coronary artery cells) stimulated with histamine, ATP, or ionomycin (6). Representative traces are shown in **Fig. 1*A***. A plateau is reached when Ca^2+^ efflux mechanisms are sufficiently stimulated to counteract Ca^2+^ influx, and hence we sought to understand how these processes contribute to altered Ca^2+^ homeostasis in cells cultured at 5% O_2_. Histamine-stimulated maximal Ca^2+^ influx measured by Ca^2+^ add-back (Fig. 1*B*, *C*) was comparable in cells adapted to 5% O_2_. Influx under more physiologic conditions (∼2 mM extracellular Ca^2+^) was assessed using the Mn^2+^ quench assay, also showing no alteration in Ca^2+^ influx capacity under 5% O_2_. Similar findings were observed in HUVECs challenged with ATP (10 µM; data not shown).

**Figure 2.**
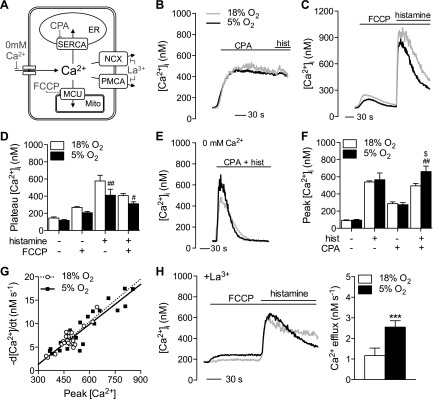
Ca^2+^ efflux pathways in ECs under 5% O_2_. HUVECs were cultured under standard (18%) or physiologic (5%) O_2_ levels, and changes in [Ca^2+^]_i_ were determined using fura-2. *A*) Schematic illustration of the Ca^2+^ efflux mechanisms present in cells and their pharmacological inhibition. FCCP indirectly inhibits the MCU by collapsing the mitochondrial membrane potential. *B*) Effects of SERCA inhibition (10 µM, CPA) on [Ca^2+^]_i_. Histamine (10 µM, hist) was added 3 min after CPA to assess the degree of store depletion. *C*) Treatment with FCCP (5 µM) alone followed by histamine stimulation as before. *D*) Mean ± sem plateau [Ca^2+^]_i_ from *C*. Treatment with CPA and/or histamine simultaneously (both 10 µM) in nominally free Ca^2+^ conditions to assess PMCA activity. *E*) Representative traces. *F*) Mean ± sem peak [Ca^2+^]_i_. *G*) Correlation between peak [Ca^2+^]_i_ after treatment with histamine and CPA and the subsequent rate of decline in [Ca^2+^]_i_ (a measure of PMCA activity). A positive correlation was confirmed by linear regression analysis, which also detected no significant difference between the 2 data sets. *H*) HUVECs were pretreated with La^3+^ (1 mM) and stimulated sequentially with FCCP (5 µM) and histamine. Representative traces and the mean rate of Ca^2+^ extrusion quantified by linear regression analysis of the slope are provided. Data denote or are representative of 4–5 different donors. ^#^*P* < 0.05, ^###^*P* < 0.01 *vs.* 18% O_2_, ***P < 0.001 *vs.* 18% O_2_, ^$^*P* < 0.05 *vs.* histamine alone.

Because the coupling between store release and Ca^2+^ influx appeared unaltered in HUVECs cultured at 5% O_2_, the lower plateau [Ca^2+^]_i_ most likely results from enhanced Ca^2+^ efflux from the cytosol. Four Ca^2+^ efflux pathways exist in ECs: plasma membrane ATPase (PMCA), SERCA, NCX, and mitochondrial Ca^2+^ uniporter (MCU). To investigate the relative contribution of SERCA and MCU, the pharmacological agents cyclopiazonic acid (CPA) and the protonophore carbonyl cyanide-4-(trifluoromethoxy) phenylhydrazone (FCCP) were used. Treatment with 10 µM CPA (16) stimulated a sustained rise in [Ca^2+^]_i_, consistent with the unopposed leakage of Ca^2+^ from the endoplasmic reticulum (ER) (**Fig. 2*B***). Further addition of histamine induced minimal additional increase. Neither the response to CPA nor subsequent addition of histamine differed significantly between O_2_ levels. In contrast, treatment with FCCP caused a small and transient increase in [Ca^2+^]_i_ that was reduced in HUVECs at 5% O_2_, as was the response to subsequent histamine stimulation (Fig. 2*C*).

Because a selective pharmacological PMCA inhibitor was not available, we used a technique described by Ferdek *et al.* (17) to investigate the role of this efflux pathway. HUVECs in nominally Ca^2+^-free buffer were cotreated with CPA (10 µM) and histamine (10 µM) to empty internal Ca^2+^ stores. With SERCA inhibited, the subsequent decline in [Ca^2+^]_i_ must represent extrusion across the plasma membrane *via* either PMCA or NCX. Because the latter is not thought to contribute to Ca^2+^ homeostasis in the absence of altered Na^+^ regulation (18) and because the Ca^2+^ response was similar when CPA was used alone (Fig. 2*B*), we did not further investigate the contribution of NCX. In contrast to individual treatments, cotreatment with CPA and histamine induced a significantly higher [Ca^2+^]_i_ release in HUVECs cultured at 5% O_2_ (Fig. 2*E*, *F*). Linear regression of the initial decline after the peak response revealed a significantly higher rate of Ca^2+^ efflux in cells at 5% O_2_. PMCA activity is proportional to [Ca^2+^]_i_ (19), and we compensated for this by plotting the rate of initial Ca^2+^ efflux against peak [Ca^2+^]_i_ (Fig. 2*G*). A positive linear correlation was observed under both O_2_ conditions. These results did not differ significantly, indicating that PMCA activity is not significantly affected at 5% O_2_. To confirm this further, HUVECs were cotreated in nominally Ca^2+^-free buffer with FCCP to inhibit mitochondrial Ca^2+^ uptake and La^3+^ to prevent extrusion across the plasma membrane (16, 20) and then treated with histamine as before (Fig. 2*H*). Under these conditions, the rate of [Ca^2+^]_i_ decline largely represents reuptake into the ER. As shown in Fig. 2*H*, Ca^2+^ extrusion was still significantly greater in cells cultured at 5% O_2_. Based on these observations, we hypothesized that increased Ca^2+^ efflux in HUVECs cultured at 5% O_2_ may be due to enhanced SERCA-mediated reuptake and not PMCA- or mitochondrial-mediated Ca^2+^ extrusion.

### SERCA activity accounts for differences in agonist-stimulated [Ca^2+^]_i_ plateau

**Figure 3.**
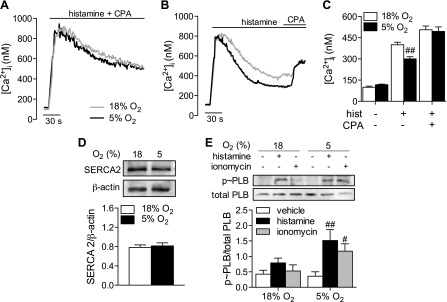
Inhibition of SERCA restores plateau [Ca^2+^] under 5% O_2_. HUVECs were cultured under standard (18%) or physiologic (5%) O_2_ levels and changes in [Ca^2+^]_i_ determined using fura-2. *A*, *B*) Histamine (10 µM) stimulated Ca^2+^ mobilization with simultaneous (*A*) or sequential (*B*) addition of CPA (10 µM). *C*) Representative traces and mean ± sem plateau [Ca^2+^]_i_ values. *D*) Expression of SERCA2 relative to β-actin. *E*) Histamine (10 µM, 30 s) and ionomycin (1 µM, 30 s) stimulated phosphorylation of PLB at S16/T17, expressed relative to total PLB levels. Representative immunoblots are shown. Densitometric analyses denote mean ± sem of measurements in 4 different donors. ^#^*P* < 0.05, ^##^*P* < 0.01 *vs.* 18% O_2_.

To confirm this hypothesis, cells were cotreated with histamine and CPA simultaneously (**Fig. 3*A***) or sequentially (Fig. 3*B*) in the presence of Ca^2+^. Histamine-mediated Ca^2+^ mobilization was similar in the presence of CPA in HUVECs at 18 or 5% O_2_. The addition of CPA during the plateau phase of histamine stimulation resulted in the same increased plateau [Ca^2+^]_i_ in cells cultured under 5% O_2_ or 18% O_2_ (Fig. 3*C*). HUVECs predominantly express the 2b isoform of SERCA when cultured, and this was unchanged after culture at 5% O_2_ (Fig. 3*D* and confirmed in Supplemental Fig. 1*C*). PLB is an endogenous regulator of SERCA2b activity, which, when bound to SERCA in its dephosphorylated state, reduces the cytosolic Ca^2+^ affinity of the pump. Phosphorylation at S16/T17 causes PLB to dissociate, thereby increasing the affinity of SERCA for Ca^2+^ (21). Although PLB is predominantly expressed in cell types such as vascular smooth muscle (22) and cardiomyocytes (23), expression of PLB has been detected in mouse aortic ECs, where it plays an important role in regulating vascular tone (24). Stimulation with histamine (10 µM) or ionomycin (1 µM) for 30 s resulted in prominent phosphorylation of PLB at S16/T17 and in a significantly higher level in cells cultured at 5% O_2_, as confirmed using immunoblotting (Fig. 3*E*) and single-cell immunofluorescence (Supplemental Fig. 1*C*). Levels of PLB phosphorylation correlated positively with SERCA2b expression in individual HUVECs (Supplemental Fig. 1*E*), and the ratio of phosphorylated ∼ PLB to SERCA2b was consistently higher in cells cultured at 5% O_2_, independent of individual cellular variance. Thus, increased PLB phosphorylation may mediate increased SERCA activity in cells under physiologic O_2_ levels.

### Enhanced SERCA activity protects against Ca^2+^-induced apoptosis

**Figure 4.**
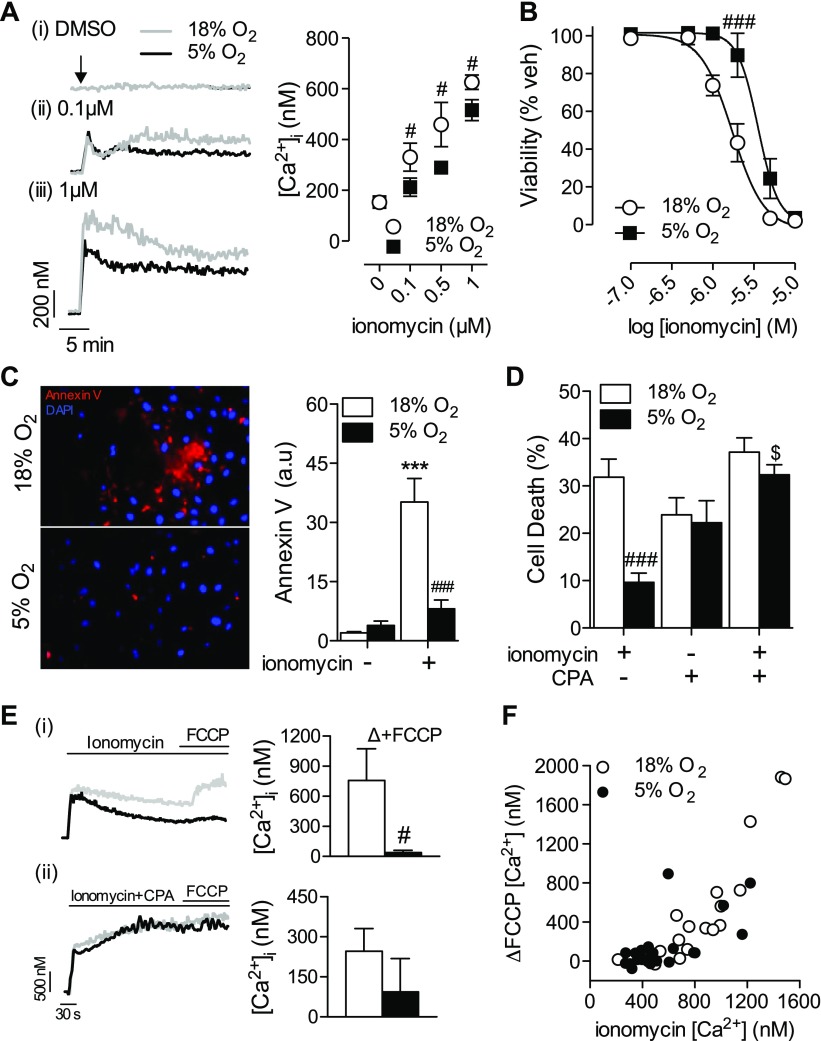
Ca^2+^ overload–induced cell death is reduced at 5% O_2_. HUVECs were cultured under standard (18%) or physiologic (5%) O_2_ levels and then stimulated with 0.1% DMSO (vehicle) or 0.1–10 µM ionomycin for 0.5–14 h. *A*) Ionomycin induced changes in [Ca^2+^]_i_. *B*) Cell death determined by MTT (2 h). *C*) Apoptosis determined by annexin V (14 h). *D*) Effects of SERCA inhibition with CPA (10 µM) in conjunction with ionomycin (0.1 µM, 14 h) on cell death. Data denote mean ± sem of measurements in 4–6 different donors. *E*) Cells were treated with ionomycin (1 µM) alone (*i*) or with CPA (*ii*) and then FCCP (5 µM) to release mitochondrial Ca^2+^. Representative traces are provided, and the mean ± sem of changes in [Ca^2+^]_i_ after FCCP treatment in ionomycin with or without CPA-treated cells is shown. *F*) The ∆[Ca^2+^]_i_ after FCCP treatment plotted against the initial plateau [Ca^2+^]_i_ after ionomycin treatment. Spearman’s rank correlation analysis revealed a strong correlation between [Ca^2+^]_i_ and FCCP-released Ca^2+^ at both 18 and 5% O_2_ (ρ = 0.91, *P* < 0.0001 and ρ = 0.62, *P* = 0.0015, respectively). ^#^*P* < 0.05, ^###^*P* < 0.001 *vs.* 18% O_2_, ^$^*P* < 0.001 *vs.* ionomycin alone, ***P < 0.001 *vs.* absence of ionomycin.

If Ca^2+^ influx largely governs cellular responses, then efflux predominantly dictates cell viability. Indeed, Ca^2+^ overload is a prominent trigger of apoptosis, as manifested during glutamate excitotoxicity in neurons (25) and in ischemia-reperfusion injury in the vascular wall and underlying parenchyma (26). Increasing Ca^2+^ reuptake capacity has been shown to ameliorate cytotoxicity caused by Ca^2+^ overload (17). Thus, one potential outcome of enhanced SERCA-mediated Ca^2+^ reuptake might be increased protection against Ca^2+^-induced apoptosis. We have used Ca^2+^ ionophore treatment as a model of Ca^2+^-induced apoptosis (27). At low concentrations, ionomycin selectively increases Ca^2+^ release from the ER without affecting membrane integrity or permeability to Ca^2+^ directly (28). As predicted, treatment with ionomycin (30 min, 0.1–1 µM) stimulated a sustained mobilization of Ca^2+^, which was significantly reduced in cells cultured at 5% O_2_ (**Fig. 4*A***). As a result, significantly less cell death was observed in HUVECs at 5% O_2_ after short-term treatment with ionomycin (0.1–10 µM, 1 h) (Fig. 4*B*), and fewer apoptotic cells were detected during more prolonged treatment (0.1 µM, 14 h) (Fig. 4*C*). To corroborate a role for SERCA in affording protection under 5% O_2_, cells were treated with ionomycin and CPA together. Similar levels of cell death were observed in response to CPA alone (10 µM, 14 h) and after cotreatment with ionomycin and CPA (Fig. 4*D*) in HUVECs cultured at 18 or 5% O_2_. Based on these observations, we hypothesized that increased SERCA activity may spare the mitochondria from excessive Ca^2^ uptake, thereby preventing the rapid initiation of the cell death cascade. The amount of FCCP-releasable Ca^2+^, an indirect measurement of mitochondrial Ca^2+^ load (29), was significantly lower after ionomycin treatment in HUVECs at 5% O_2_ (Fig. 4*E**i*). Cotreatment with ionomycin and CPA significantly increased [Ca^2+^]_i_ under both O_2_ levels and to a similar level (Fig. 4*E**ii*), and subsequent addition of FCCP had no further effect, probably reflecting either the limits of detection for Fura-2 or a complete breakdown in Ca^2+^ homeostasis. A correlation was observed between the plateau [Ca^2+^]_i_ after stimulation with ionomycin and the amount of FCCP-releasable Ca^2+^ (Fig. 4*F*). This correlation strongly corroborates our hypothesis that reducing cytosolic [Ca^2+^]_i_ *via* increased SERCA activity can indirectly decrease mitochondrial Ca^2+^ uptake.

## DISCUSSION

The biologic importance of Ca^2+^ clearance is underpinned by the significant energy expended in maintaining the >10,000-fold concentration gradient between cytosolic and extracellular/store [Ca^2+^]. Perturbations in these processes are a hallmark of numerous pathophysiological conditions and are ultimately a key trigger of cell death (21). Hence, our finding that Ca^2+^ clearance is enhanced when ECs are cultured under their physiologic O_2_ level is important. Increasing evidence suggests that the culture of mammalian cells at room air (∼18% O_2_) robustly alters cellular phenotype, which may account for artifactual findings. For example, many primary human cell types show pronounced senescence or apoptosis during routine cell culture, which can be attributed to hyperoxia and the overproduction of reactive oxygen species associated with elevated O_2_ levels (30). In ECs, adaptation to this hyperoxic environment *in vitro* results in exacerbated nuclear factor E2-related factor 2–regulated antioxidant defense (4), less efficient eNOS dephosphorylation (6), and reduced barrier integrity (5). The present study provides the first characterization of the mechanisms underlying Ca^2+^ dysregulation during long-term culture under hyperoxic conditions.

The fact that histamine-stimulated peak [Ca^2+^]_i_ was unaltered at 5% O_2_ in the presence or absence of external Ca^2+^ (Fig. 1) indicates that the coupling between receptor occupancy and Ca^2+^ store release is not affected. Instead, the ability of SERCA to resequester Ca^2+^ into the ER appears to be enhanced in cells at an O_2_ level more akin to *in vivo* conditions. Ca^2+^ add-back and Mn^2+^ quench assays (Fig. 1*B*, *C*) suggested no change in total store-operated Ca^2+^ influx capacity. However, actual influx may differ under sustained stimulation due to the coupling between Ca^2+^ influx and ER store content because the latter may alter because of increased SERCA activity. Unlike the actions of PMCA or NCX, in which Ca^2+^ is extruded from the cell completely, SERCA-mediated Ca^2+^ clearance only solves half the problem because low cytosolic Ca^2+^ is maintained at the expense of increased stored intracellular Ca^2+^. Accounting for this increased load at 5% O_2_ could involve either enhanced shuttling between the ER and mitochondria (31) or an increased buffering capacity of the ER. The former seems unlikely based on our observations that the mitochondria are less loaded with Ca^2+^ in cells at 5% O_2_ (Figs. 2*C* and 4*E*) and the knowledge that mitochondrial Ca^2+^ uptake can potentiate Ca^2+^-induced cell death (32). Instead, our observation that Ca^2+^ release after histamine stimulation in nominally Ca^2+^-free buffer was significantly higher when SERCA was inhibited only at 5% O_2_ (Fig. 2*E*, *F*) corroborates the observation of an increase in total stored Ca^2+^ in these cells (33). Pretreatment with FCCP tended to abolish this difference in peak [Ca^2+^]_i_ release by CPA + histamine in nominally Ca^2+^-free buffer (data not shown), suggesting the mitochondria may play a subtle role in ER Ca^2+^ homeostasis under nonphysiologic conditions. Enhanced ER capacity may buffer elevations in mitochondrial Ca^2+^, although increased Ca^2+^ storage in the ER has also been shown to reduce cell viability under pathologic conditions (34).

ECs usually express SERCA2b *in vivo* and *in vitro*, and some also express SERCA3 (35–37). HUVECs are unique in that they solely express SERCA3 *in vivo* (36), yet this diminishes rapidly after culture. We view this as fortuitous because it results in a phenotype more consistent with the general endothelium, and we posit that their distinctive expression pattern may reflect the unique *in vivo* umbilical environment. SERCA2b is susceptible to regulation by PLB and has a 5- to 10-fold higher affinity for Ca^2+^ than SERCA3, which is not PLB regulated (38, 39). Thus, under conditions in which both isoforms are expressed, SERCA2b plays the predominant role in regulating cytosolic Ca^2+^. Aortic rings isolated from PLB knockout mice exhibit diminished endothelium-dependent relaxation to acetylcholine (24), and, because SERCA3 is insensitive to PLB, this implies a functional role for SERCA2b in ECs *in vivo*. Our findings demonstrate limited PLB phosphorylation in HUVECs cultured in room air (Fig. 3*E*), suggesting that this pathway is down-regulated in cells adapted to standard hyperoxic culture conditions. Increased NO availability has been linked to enhanced Ca^2+^ uptake (40), with both protein kinase G (22) and AMP-activated protein kinase (41) having been shown to target PLB to alleviate its inhibitory influence on SERCA. Thus, enhanced SERCA activity in cells cultured at 5% O_2_ may be linked to favorable NO bioavailability under these conditions (6). Increased PLB phosphorylation at 5% O_2_ (Fig. 3*E*) may enhance SERCA Ca^2+^ affinity and thereby enable more rapid activation in response to Ca^2+^ release, limiting complete store emptying in response to histamine alone. Although 75–90% of HUVECs in a given population exhibit functional PLB phosphorylation (Supplemental Fig. 1*B*), total expression is still severalfold lower than that observed in cardiomyocytes (41). Moreover, differences in the percentage of cells expressing both SERCA and p ∼ PLB (77% at 18% O_2_ *vs.* 90% at 5% O_2_; Supplemental Fig. 1*B*) may offer an additional explanation for the altered Ca^2+^ homeostasis and susceptibility to Ca^2+^ overload under physiologic normoxia. Furthermore, our data cannot discount the possibility of heterogeneous coupling of SERCA-PLB at the cellular level, although it is unlikely that this hypothesis could explain the protection afforded by culture under physiologic normoxia.

As we recently demonstrated (6), key Ca^2+^-dependent processes such as eNOS activation are not reduced in HUVECs cultured at 5% O_2_, despite reductions in plateau [Ca^2+^]_i_. This likely reflects the fact that most of these processes are dependent on Ca^2+^ influx and not on net [Ca^2+^] (42). Moreover, increased calmodulin expression in HUVECs at 5% O_2_ (6) may compensate for reduced [Ca^2+^]_i_. Rather, Ca^2+^ clearance efficiency can become an important factor in maintaining cellular function during episodes of prolonged [Ca^2+^]_i_ elevation (26). Under these conditions, the ability of the cell to effectively lower cytosolic Ca^2+^ can ultimately determine cell survival. We have demonstrated that increased SERCA activity can protect HUVECs against ionomycin-induced Ca^2+^ overload at 5% O_2_ (Fig. 4). Increased apoptosis/cell death is often observed in cells with dysfunctional Ca^2+^ ER homeostasis (33) or Ca^2+^ extrusion mechanisms (17). Our results indicate that, in the absence of preexisting pathology, increased Ca^2+^ reuptake may protect cells against acute Ca^2+^ overload. Prolonged culture under hyperoxic conditions detrimentally affects Ca^2+^ homeostasis, ultimately sensitizing cells toward damaging stimuli. We therefore propose that culture under physiologic O_2_ levels may provide a more suitable *in vitro* model to investigate treatments aimed at alleviating dysregulation of Ca^2+^ handling.

## Supplementary Material

This article includes supplemental data. Please visit *http://www.fasebj.org* to obtain this information.

## Abbreviations

CPA: cyclopiazonic acid
EC: endothelial cell
ER: endoplasmic reticulum
FCCP: carbonyl cyanide-4-(trifluoromethoxy) phenylhydrazone
MCU: mitochondrial Ca^2+^ uniporter
MTT: 3-(4,5-dimethylthiazol-2-yl)-2,5-diphenyltetrazolium bromide
NCX: Na^+^:Ca^2+^ exchanger
PLB: phospholamban
PMCA: plasma membrane Ca^2+^ ATPase
SERCA: sarco/endoplasmic reticulum Ca^2+^ ATPase
