# Supplementary material for: Reduced SERCA activity underlies dysregulation of Ca2+ homeostasis under atmospheric O2 levels
Source: FASEB J. 2018 Jan 8;32(5):2531–8. doi: 10.1096/fj.201700685RRR (PMC5901376; doi:10.1096/fj.201700685RRR)
Supplement: Supplementary file 1 [file fj.201700685RRR.sf1.pdf]

Supplementary Data Unit 1

Methods

Immunofluorescent quantification of SERCA2 and p~PLB expression

Cells were cultured on 8-well coverslips, fixed using 4% paraformaldehyde and blocked in 5% bovine serum albumin. Sections were stained with anti-SERCA2 (IID8) and anti-phospho (S16/T17) phospholamban (Abcam, UK) and associated fluorescently-tagged secondary antibodies. Cells were visualised with a Nikon Diaphot microscope and imaged using an ORCA-03G camera. DAPI fluorescence was used to create a nuclear mask and any fluorescence in SERCA/p~PLB images contained within this were subtracted from the whole cell fluorescence, before correcting for background fluorescence. Expression of a protein (as in Figure S1B) was defined as a resulting mean grey value 5% above the background.

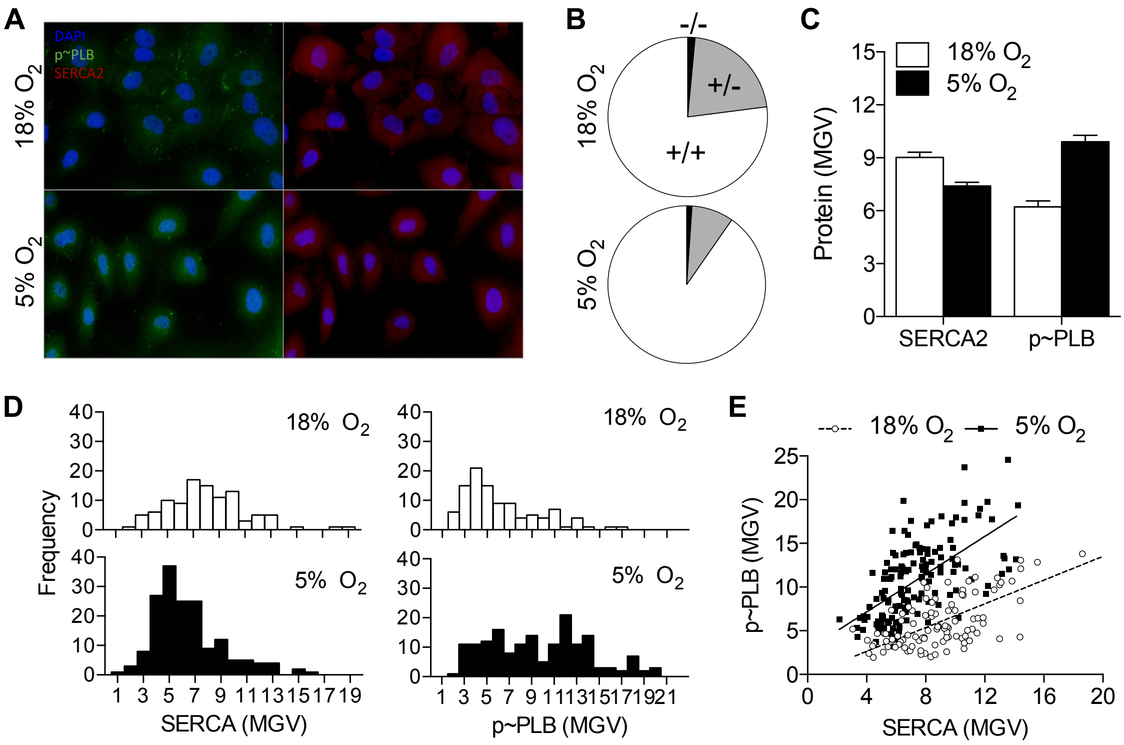

Supplementary Figure 1. Single cell analysis of SERCA2 and phosphorylated phospholamban expression. HUVEC were cultured under standard (18%) or physiological (5%) O_2 levels, stimulated with 10µM histamine for 1 minute then fixed and stained with anti-SERCA2 and anti-phospho(S16/T17) phospholamban antibodies and associated fluorescently-tagged secondary antibodies, with DAPI used to counterstain the nuclei. (**A**) Representative images, (**B**) Percentage of cells expressing both SERCA and p~PLB (+/+), only SERCA (+/-) or neither proteins (-/-), determined by thresholding mean grey values 5% above background. Only those cells expressing both proteins were used for subsequent analysis. (**C**) Mean expression of SERCA and p~PLB, and (**D**) frequency distribution of SERCA and p~PLB expression. (**E**) The relationship between p~PLB and SERCA expression in individual cells. Linear regression analysis reveals a positive linear correlation (r^2=0.43 and 0.38 at 18% and 5% O_2, respectively) and a significantly greater slope at 5% O_2 (0.67±0.08 vs 1.08±0.11, P<0.01). Data are taken from 3 independent wells of cells from a single donor.
